# Mediastinal Lymphadenopathy Presenting With the "Medu Vada Sign"

**DOI:** 10.7759/cureus.86939

**Published:** 2025-06-28

**Authors:** Yuvarajan Sivagnaname, Praveen Radhakrishnan, Durga Krishnamurthy, Navya Cherukkumalli, Sagana Ravikumar

**Affiliations:** 1 Department of Respiratory Medicine, Sri Manakula Vinayagar Medical College and Hospital (SMVMCH), Puducherry, IND; 2 Department of Obstetrics and Gynaecology, Sri Lakshmi Narayana Institute of Medical Sciences, Puducherry, IND

**Keywords:** doughnut sign, ebus, mediastinal adenopathy, medu vada, tb

## Abstract

The "Medu Vada" (or "doughnut") sign is a compelling radiological finding often noted on axial chest CT scans, characterized by a circular or ring-like appearance. This distinct morphology arises from enlarged mediastinal adenopathy, specifically involving lymph nodes such as those in the subcarinal or right paratracheal areas, which encircle the adjacent airway or esophagus. We report the case of a 55-year-old female who presented with a constellation of non-specific constitutional symptoms, including chronic cough, significant weight loss, diminished appetite, and recurrent evening fevers. Diagnostic imaging revealed extensive mediastinal adenopathy that bore a striking resemblance to the classic "Medu Vada" configuration. Subsequent investigations confirmed a diagnosis of tuberculous lymphadenitis. This case highlights the importance of recognizing this specific imaging pattern, which we propose to formally name the "Medu Vada sign," as it can serve as a valuable clue in the differential diagnosis of mediastinal pathology, particularly in areas where tuberculosis is endemic.

## Introduction

Mediastinal lymphadenopathy, defined as the enlargement of lymph nodes within the central thoracic compartment, is a common radiological finding with diverse etiologies, including infections such as tuberculosis, inflammatory conditions like sarcoidosis, and malignant processes like lymphoma or metastases. Accurate differentiation among these causes is crucial, as it directly influences treatment decisions and prognosis. Although histopathological analysis is the gold standard for diagnosis, specific radiological signs can significantly guide clinicians in their assessment. In this context, we describe the "Medu Vada" sign (doughnut sign on the chest), a distinctive radiological appearance characterized by a ring-like configuration of mediastinal lymph nodes surrounding the airway on contrast-enhanced CT imaging. This sign, reminiscent of the South Indian lentil fritter "Medu Vada," reflects the ring-like lymph node configuration around a central airway.

## Case presentation

A 55-year-old female presented to the hospital with a two-month history of a dry cough, low-grade intermittent fever (predominantly in the evenings), and unexplained weight loss. Additionally, she reported chest pain for the past 15 days. On clinical examination, her vital signs were stable, and a thorough systemic examination did not reveal any obvious clinical signs or clues that suggested a specific underlying pathology. Initial laboratory investigations revealed an elevated erythrocyte sedimentation rate (ESR) and C-reactive protein (CRP) level, both of which are indicative of an inflammatory pathology. Complete blood counts (CBC) were within normal limits (Table 1).

**Table 1:** Hematological and Biochemical Investigations

Investigation	Patient Value	Normal Range
Erythrocyte Sedimentation Rate (ESR)	56 mm/hour	0–20 mm/hour (males)/0–30 mm/hour (females)
C-Reactive Protein (CRP)	38 mg/L (elevated)	<6 mg/L
Hemoglobin	13.6 g/dL	13–17 g/dL (males)
Total Leukocyte Count	7,800/µL	4,000–11,000/µL
Platelet Count	2.4 lakh/µL	1.5–4.0 lakh/µL
HIV 1 & 2 Serology	Non-reactive	Non-reactive
Mantoux Test (Tuberculin Skin Test)	15 mm induration	<10 mm (negative)
Serum Angiotensin-Converting Enzyme (ACE)	68 U/L	8–52 U/L
Serum Calcium	9.4 mg/dL	8.5–10.5 mg/dL
Serum Albumin	4.2 g/dL	3.5–5.0 g/dL
Alanine Transaminase (ALT)	28 U/L	10–40 U/L
Serum Creatinine	0.9 mg/dL	0.6–1.3 mg/dL
Blood Urea	28 mg/dL	10–40 mg/dL
Serum Sodium	138 mmol/L	135–145 mmol/L
Serum Potassium	4.2 mmol/L	3.5–5.1 mmol/L
Total Protein	7.0 g/dL	6.0–8.3 g/dL
Serum Globulin	2.8 g/dL	2.0–3.5 g/dL
Random Blood Glucose	96 mg/dL	70–140 mg/dL
HbA1c	5.4%	<5.7% (normal)

**Figure 1:**
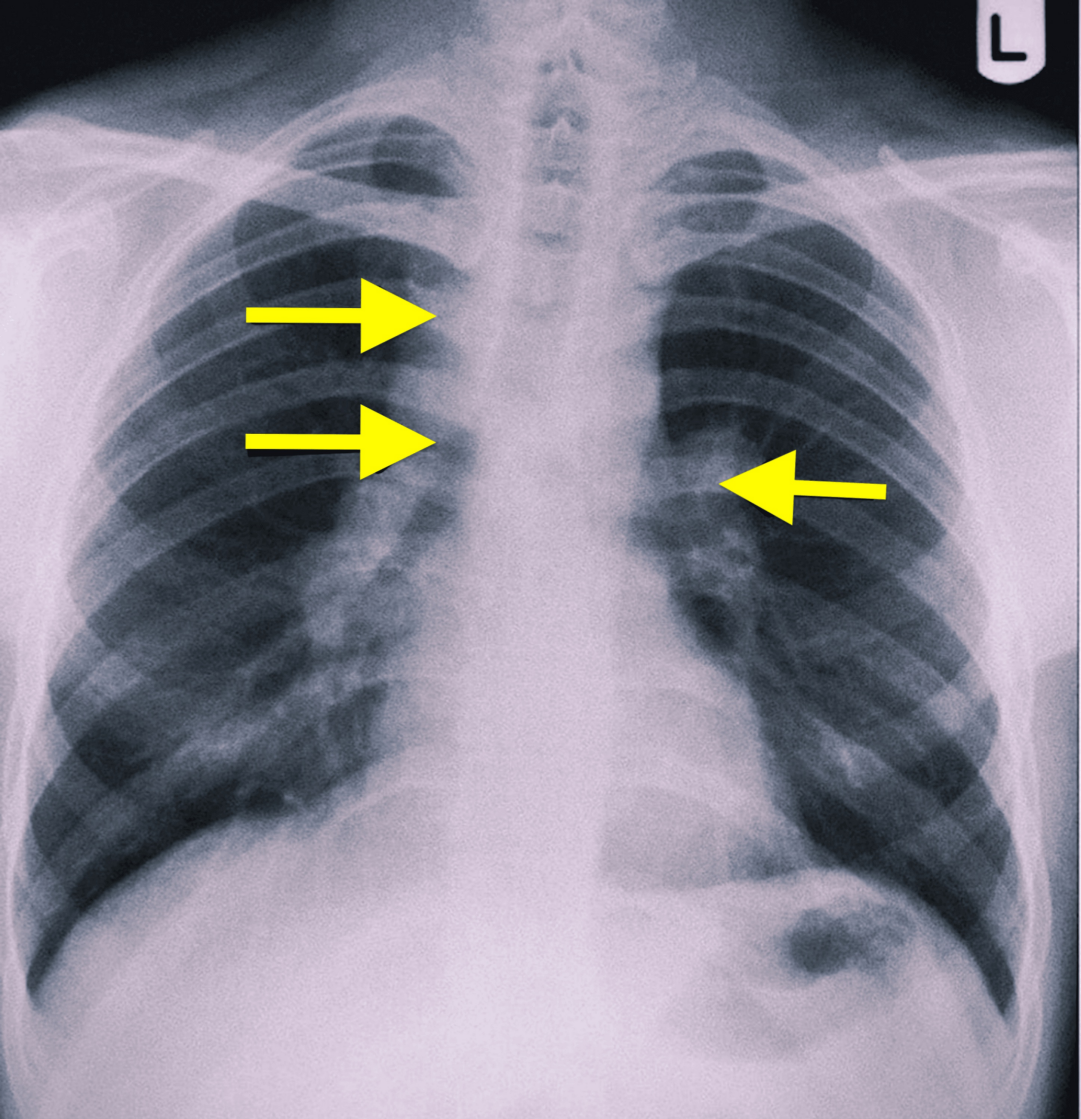
Chest radiograph posteroanterior view showing bilateral hilar and right paratracheal adenopathy

A chest radiograph was done, which showed bilateral hilar prominence with right paratracheal widening (Figure 1).

**Figure 2:**
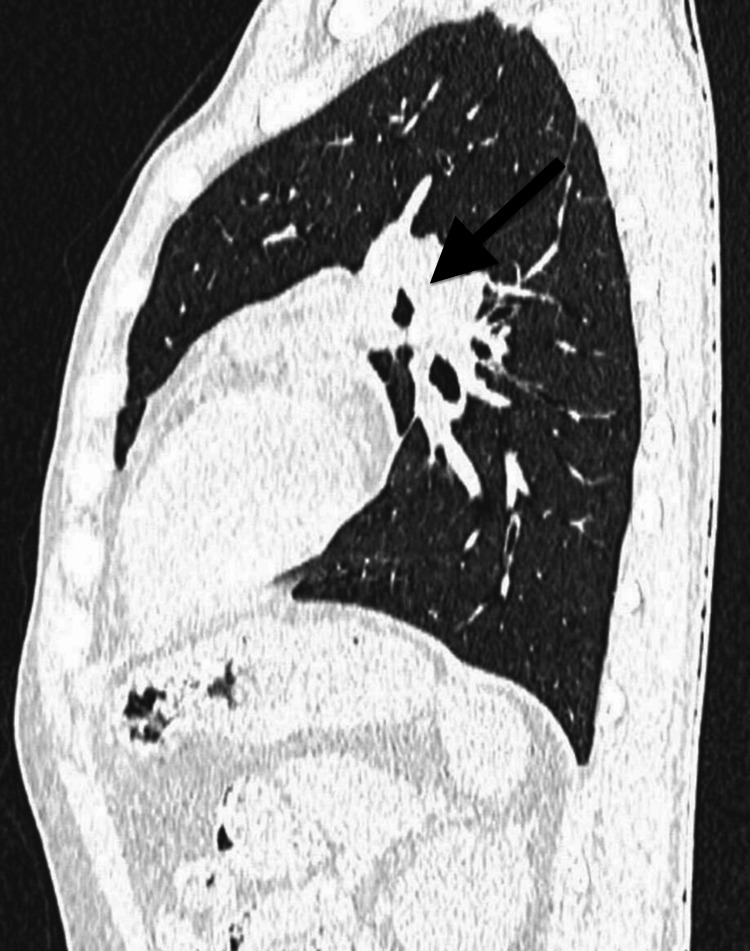
Computed tomogram of the chest sagittal view (lung window) showed conglomerated nodes forming a ring-like configuration around the airway, strikingly resembling a "Medu Vada" (doughnut)

**Figure 3:**
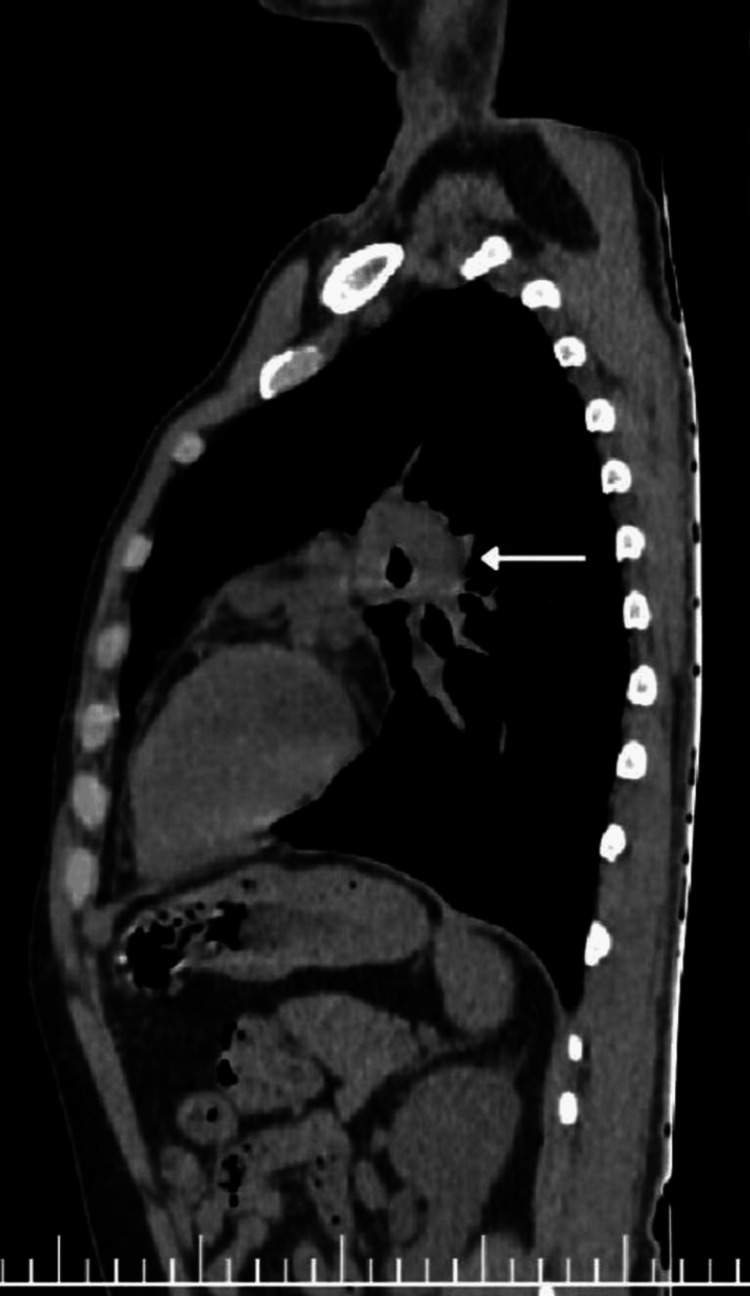
Sagittal view (mediastinal window) showing the "Medu Vada" sign

Computed tomography of the thorax provided more detailed anatomical insights. It revealed enlarged right paratracheal and subcarinal lymph nodes, which appeared conglomerated. Crucially, these nodes demonstrated a characteristic pattern of central hypodensity with peripheral enhancement. The sagittal view of the lung and mediastinal window distinctly showed a ring-like configuration, strikingly resembling a "doughnut" or, more specifically, the "Medu Vada" (Figures 2, 3).

Importantly, no cavitary lesions or active parenchymal disease were noted in the lung fields. The radiological impression was mediastinal lymphadenopathy with central necrosis, with the "Medu Vada" sign explicitly indicated as positive, strongly suggesting tuberculous lymphadenitis as the most likely etiology.

**Figure 4:**
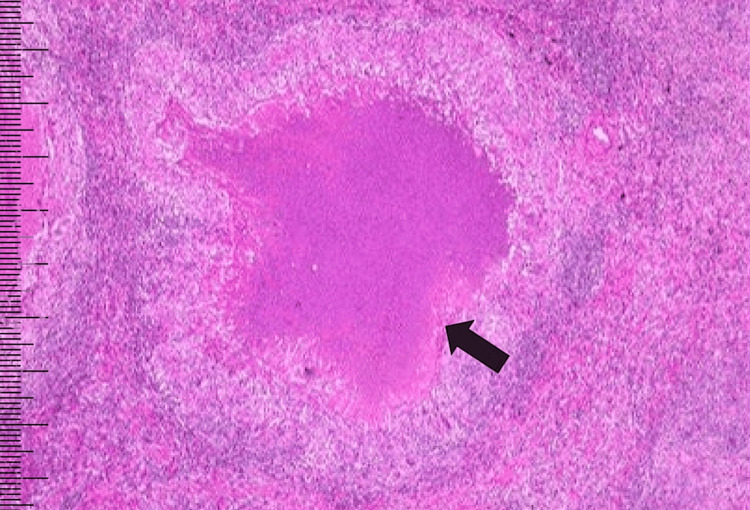
EBUS aspirate from the subcarinal node showing epithelioid granuloma with caseous necrosis (H&E stain, 20×) EBUS: endobronchial ultrasound; H&E: hematoxylin and eosin

Based on the clinical presentation and initial imaging findings, tuberculous mediastinal lymphadenitis, sarcoidosis, and lymphoma were considered as differential diagnoses. To establish a definitive diagnosis, an endobronchial ultrasound (EBUS)-guided fine needle aspiration (FNA) was performed on a subcarinal lymph node. Histopathological examination of the aspirate confirmed the presence of caseating granulomas with necrosis, a hallmark feature of tuberculosis (Figure 4).

While the acid-fast bacilli (AFB) direct smear was negative, subsequent molecular testing using GeneXpert (Cepheid, Sunnyvale, CA, USA) detected *Mycobacterium tuberculosis*, with no resistance to rifampicin. This comprehensive diagnostic workup unequivocally confirmed the final diagnosis. The final diagnosis for the patient was mediastinal tuberculous lymphadenitis presenting with the "Medu Vada" sign.

Treatment and outcome

The patient was promptly initiated on standard anti-tubercular therapy (ATT). This regimen included an intensive phase of HRZE (isoniazid, rifampicin, pyrazinamide, and ethambutol) for two months, followed by a continuation phase of HRE (isoniazid, rifampicin, and ethambutol) for four months. After two months of ATT, the patient showed significant symptomatic improvement, including weight gain. A repeat CT imaging performed at the completion of the ATT course demonstrated a significant reduction in lymph node size and the disappearance of the "Medu Vada" appearance, further confirming the successful resolution of the tuberculous lymphadenitis.

## Discussion

This case highlights the diagnostic relevance of the "Medu Vada" sign (doughnut sign on the chest) in tuberculous mediastinal lymphadenopathy. On CT, tuberculous lymph nodes may demonstrate central low attenuation with peripheral rim enhancement [1-4]. However, the defining feature of the "Medu Vada" sign (doughnut sign on the chest) is the morphological pattern of lymph nodes encircling the airway, rather than the internal architecture or necrosis. The nodal arrangement surrounding the tracheobronchial tree forms a ring-like pattern that radiologically mimics the Indian snack "Medu Vada," offering a culturally relevant analogy.

The sign reflects the confluence of enlarged lymph nodes that circumferentially outline the airway, creating a radiological pattern that draws diagnostic attention. This specific configuration may result from nodal conglomeration or matting, which are more frequently associated with infectious or inflammatory etiologies, especially tuberculosis [1,5]. In contrast, sarcoidosis typically presents with bilaterally symmetrical, discrete lymphadenopathy, while lymphoma often manifests as large, homogeneous masses without airway encasement [6,7].

The cultural relevance of the "Medu Vada" sign (doughnut sign on the chest) is particularly significant in South Asian populations, where the "Medu Vada" is a widely recognized culinary item. Compared to the traditional term "doughnut sign," the "Medu Vada" analogy is more relatable and potentially more accurate in describing the typically irregular, variably lobulated appearance of conglomerated lymph nodes. Unlike the uniformly shaped doughnut, a "Medu Vada" has a slightly more variable and lobular contour, closely resembling the actual CT morphology of tuberculous lymphadenopathy.

Moreover, this terminology enhances retention and recall among clinicians and radiologists, especially in regions with a high prevalence of tuberculosis. The simplicity and familiarity of the analogy can improve teaching utility and communication in both clinical and academic settings. When correlated with suggestive clinical features and supported by diagnostic modalities such as EBUS-FNA and CBNAAT (cartridge-based nucleic acid amplification test) [8,9], the identification of the "Medu Vada" sign (also known as the doughnut sign on the chest) can expedite diagnosis and treatment.

Importantly, the presence of this sign should prompt clinicians to consider tuberculosis as a key differential, particularly in endemic areas, and prioritize early diagnostic confirmation through tissue sampling. The resolution of the sign following ATT, as seen in our case, also indicates its potential as a marker of therapeutic response [2-4,10].

## Conclusions

The "Medu Vada" sign (doughnut sign on the chest) is a visually distinctive and diagnostically useful radiological sign suggesting a ring-like configuration of mediastinal lymph nodes surrounding the airway, often seen in tuberculous pathology. In endemic areas, its recognition should raise suspicion for tuberculosis and prompt confirmatory diagnostic steps. Integration of this sign into radiological practice can aid in early diagnosis, effective treatment planning, and monitoring of therapeutic response.
